# Patterns of late gadolinium enhancement in 94 patients with AL or transthyretin cardiac amyloidosis

**DOI:** 10.1186/1532-429X-14-S1-O87

**Published:** 2012-02-01

**Authors:** Jason Dungu, Carol J Whelan, Simon D Gibbs, Jennifer H Pinney, Sanjay M Banypersad, Christopher P Venner, Helen J Lachmann, Ashutosh Wechalekar, Julian D Gillmore, Philip N Hawkins, Lisa Anderson

**Affiliations:** 1 UK National Amyloidosis Centre, London, UK; 2 St George's University of London, London, UK

## Background

Cardiac MRI (CMR) is increasingly used to further investigate patients in whom amyloidosis is suspected on echocardiography. Late gadolinium enhancement (LGE) reflects expansion of the interstitium, and circumferential subendocardial LGE has been reported to be a typical finding in AL amyloidosis; by contrast, a more diffuse transmural LGE pattern has been associated with ATTR (transthyretin amyloidosis).

## Methods

We studied patients who had been referred to the UK National Amyloidosis Centre (NAC) between June 2007 and June 2011 with a possible diagnosis of cardiac amyloidosis based on the findings of CMR performed at their local centres. We analysed the CMR images and LGE patterns in the left ventricle at the base, mid wall and apex. The LGE pattern at each level was described as circumferential if it involved all segments at either the base, mid or apical levels, and transmural if it involved the subendocardium through to the epicardial layer.

## Results

Studies were performed in 32 UK centres, and we were able to obtain 103 studies for analysis. The mean age of the patients was 69.2 ± 11.4 years with a male predominance (72.7%). Amyloidosis was confirmed at the NAC in 99 patients and excluded in 4 patients. The amyloid type was AL in 39 patients (39.4%), ATTR in 55 patients (55.5%) and rarer forms of non-AL, non-TTR amyloid in 5 patients (5.1%). We report the CMR findings in 94 patients with AL or ATTR. Interventricular wall thickness was greater in ATTR compared to AL (16.7 mm ± 2.5 vs 14.2 mm ± 2.1, p <0.01). LGE was evident in all patients with ATTR amyloid and 36 patients (92.3%) with AL (p=0.07). Right ventricular (RV) LGE was also present in all patients with TTR amyloid and 26 patients (66.7%) with AL (p <0.01). A gradient with greater LGE at the base than the apex was more often present in ATTR (62.5% vs 35.9% in AL - p <0.01). Circumferential subendocardial LGE was present in only 8 patients (20.5%) with AL and only 2 patients (3.6%) with ATTR (p <0.01). Circumferential transmural LGE was present in 6 patients with ATTR (10.7%) and none with AL amyloid (p<0.01). Fifty-two patients with ATTR (92.9%) demonstrated transmural LGE in at least one segment compared to 12 patients (30.8%) with AL amyloid (p <0.01).

## Conclusions

This study of CMR performed in multiple regional centres confirms its high diagnostic value in cardiac amyloidosis. Whilst greater wall thickness and a transmural pattern of LGE was more suggestive of ATTR, only the absence of RV LGE in one-third of AL patients reliably distinguished the two types of amyloid.

## Funding

Funding for this study was obtained from the British Heart Foundation Clinical Research Training Fellowship no. FS/09/063/28026 to J.D.

**Table 1:** Comparison of the cardiac MRI features in patients with AL and TTR amyloidosis.

	AL (n=39)	ATTR (n=55)	P value
Age (years)	64.3 ± 9.6	74.1 ± 8.6	<0.01
Survival (months)	18	48	<0.01
NT pro-BNP (pMol/L)	644 (84-7323)	497 (43-2791)	0.177
Interventricular septum (mm)	14.2 ± 2.1	16.9 ± 2.2	<0.01
Late gadolinium enhancement (LGE)	36 (92.3%)	55 (100%)	0.07
RV LGE	26 (66.7%)	55 (100%)	<0.01
Circumferential subendocardial LGE	8 (20.5%)	2 (3.6%)	0.01
Circumferential transmural LGE	0 (0%)	6 (10.9%)	<0.01
Base-apex gradient	14 (35.9%)	35 (63.6%)	0.01
Any segment transmural LGE	12 (30.8%)	52 (94.5%)	<0.01

**Figure 1:**
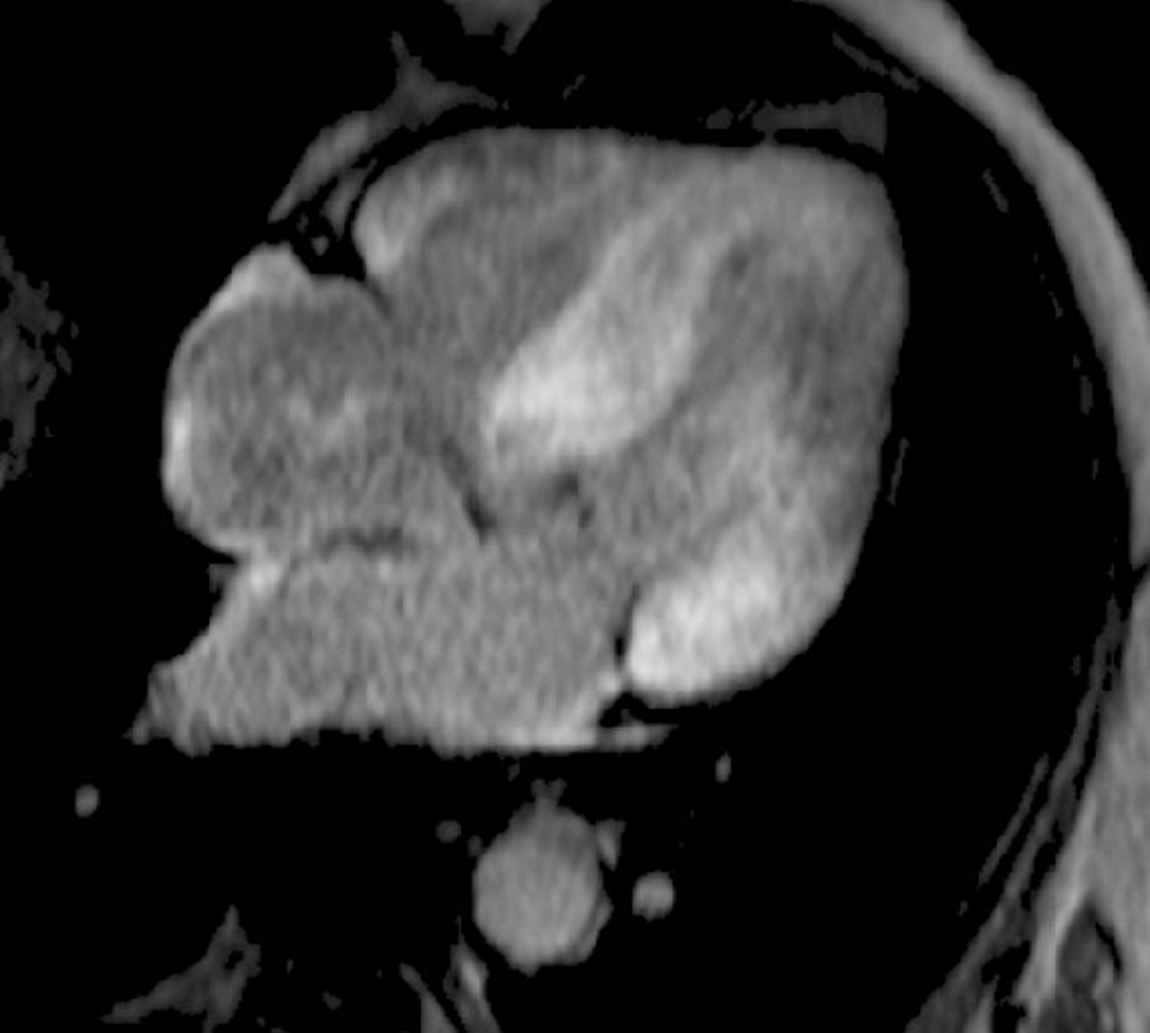
A late gadolinium enhancement image in a patient with ATTR demonstrating biventricular thickening, transmural enhancement at the base, a base-apex gradient and right ventricular involvement.

